# Urgent Transcatheter Mitral Valve-in-Valve Replacement With Venoarterial Extracorporeal Membrane Oxygenation Support: Case Report and Review of the Literature

**DOI:** 10.7759/cureus.52920

**Published:** 2024-01-25

**Authors:** Mohammad Abdel Jawad, Cyrus M Munguti, Abdullah Abu Kar, Venkata Boppana, Zaher Fanari

**Affiliations:** 1 Internal Medicine, Ascension Via Christi St. Francis, Wichita, USA; 2 Internal Medicine, University of Kansas, Wichita, USA; 3 Hospital Medicine, University of California, San Francisco, USA; 4 Cardiology, Heartland Cardiology, Wichita, USA; 5 Cardiology, University of Kansas, Wichita, USA; 6 Cardiology, University of California San Francisco, Fresno, USA

**Keywords:** severe mitral stenosis, cardiogenic shock, failed bioprosthetic valves, mechanical circulatory support, mitral valve-in-valve replacement

## Abstract

Critical mitral valve stenosis due to a failed bioprosthetic valve is associated with significant morbidity and mortality, with the transcatheter Valve-in-Valve (ViV) approach becoming a popular treatment option. We present a case of cardiogenic shock secondary to a stenotic mitral bio-prosthetic valve. The Heart team was consulted; the patient was a high-risk surgical candidate for valve replacement. He required venoarterial extracorporeal membrane oxygenation as a bridge to definitive therapy. The patient underwent a successful urgent transcatheter mitral ViV procedure with a trans-septal approach. Follow-up echocardiography showed significant improvement in mitral valve dynamics. Recently emerging transcatheter approaches for mitral ViV implantation after balloon valvuloplasty into a failed mitral valve prosthesis are technically feasible in high-risk patient populations and should be considered over re-operative mitral valve surgery.

## Introduction

Mitral valve disease is one of the most common valvular heart diseases, with more than 20,000 mitral valve replacement procedures performed each year in the US [1]. Due to their limited durability, reoperation on bioprosthetic mitral valves is an unavoidable trajectory in one-third of patients and is associated with significant morbidity and mortality [2]. To mitigate the high surgical risk associated with reoperation, the mitral Valve-in-Valve (ViV) concept was introduced in 2007 and showed favorable results [3]. Transcatheter mitral ViV is associated with low complication rates and lower-than-predicted mortality rates [4]. Here, we describe a case of severe mitral stenosis in a patient with a failed bioprosthetic mitral valve, resulting in cardiogenic shock requiring venoarterial extracorporeal membrane oxygenation (VA-ECMO). The patient underwent a successful urgent transcatheter transseptal mitral ViV procedure.

This case was presented as a complex clinical case abstract at the American College of Cardiology Annual Scientific Meeting in April 2022.

## Case presentation

**Figure 1:**
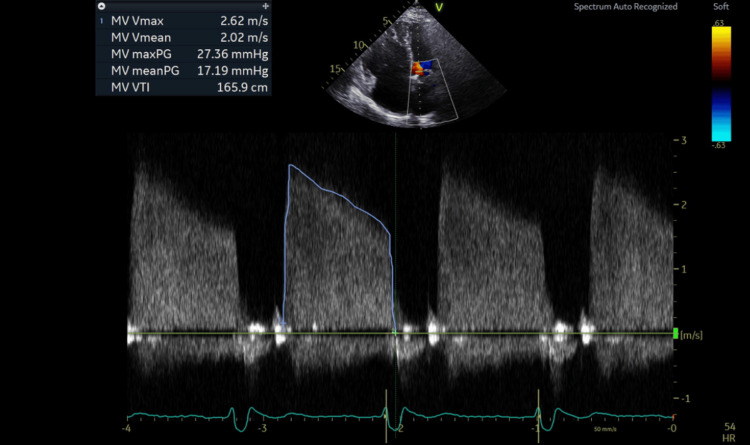
Spectral Doppler waveform prior to mitral valve in valve replacement. Mean pressure gradient across the mitral valve is 17.19 mmHg indicating severe mitral stenosis.

A 73-year-old male with medical history of atrial fibrillation, congestive heart failure, hypertension, peptic ulcer disease, chronic kidney disease stage III, and nonrheumatic mitral stenosis status post bioprosthetic mitral valve replacement (MVR) x2, in 2015 using a 27 mm bioprosthetic valve (Medtronic, Minneapolis, MN) and redo MVR in 2017 using a 25 mm bioprosthetic valve (St. Jude Medical, St. Paul, MN). The patient is active at baseline with a New York Heart Association (NYHA) functional class of II. The patient presented to our hospital with 1 week of worsening shortness of breath and lack of energy. His admission to NYHA functional Class was III. During the physical exam, the heart rate was 65 beats per minute, the respiratory rate was 22 breaths per minute, the oxygen saturation was 85% while breathing ambient air, and the blood pressure was 116/61 mmHg. The patient was tachypneic but not in acute distress. Examination of the heart and lungs revealed an apical diastolic murmur, bibasilar crackles, and trace leg edema. Abnormal labs on admission include a creatinine of 1.74 (baseline 1.3 mg/dL) and a brain natriuretic peptide (BNP) of 2416 pg/ml (reference range < 100). Chest x-ray on admission showed mild cardiomegaly and pulmonary venous congestion. Transthoracic echocardiogram (TTE) showed an ejection fraction of 75-80%, a bioprosthetic mitral valve with critical mitral stenosis, and moderate mitral regurgitation (Figure 1, Video 1).

**Video 1:** Transthoracic echocardiography showing severe prosthetic mitral valve stenosis.

The patient was admitted to the medical floor and was started on intravenous diuretics with furosemide. The patient's clinical condition deteriorated over the following few days; his oxygen requirement increased from 2L on admission to requiring a heated high-flow nasal cannula. He also developed cardiogenic shock, requiring transfer to the intensive care unit. His blood pressure was closely monitored via an arterial line in the right brachial artery, as the Swan-Ganz catheter was not used (unfortunately, this was not an option for this patient due to technical issues). The patient was started on vasopressors and inotropic therapy (norepinephrine, vasopressin, and dobutamine). The patient's Society of Cardiovascular Angiography and Interventions (SCAI) shock grade was D. His cardiogenic shock was complicated with shock liver, worsening hypoxia, coagulopathy, and worsening kidney function. The patient's condition continued to deteriorate, and so did his oxygen requirement, so he was intubated and started on VA-ECMO through a left femoral access. The patient was started on argatroban for anticoagulation; heparin products were not used due to a concern about heparin-induced thrombocytopenia. The heart team consisted of structural cardiology and cardiothoracic surgery, evaluated the patient, and decided to perform an urgent mitral ViV procedure. He was not a candidate for a third redo mitral valve surgery due to extremely high surgical risk, with a society of thoracic surgeons predicted risk of mortality score of 28% with combined mortality and morbidity of 87%. Pre-procedural chest computed tomography angiography showed a bioprosthetic mitral valve in place with mild noncalcified thickening of the leaflets. The TTE conducted after the patient's admission to the hospital revealed severe bioprosthetic mitral valve stenosis. It was also noted that the patient had a history of severe bioprosthetic mitral valve stenosis from previous studies and follow-ups with our clinic. Therefore, we did not deem it necessary to perform transesophageal echocardiography (TEE) as we believed it would not add any value to the diagnosis. However, a TEE was performed during the ViV procedure.

The patient was brought to a hybrid room. Access was obtained through the right groin. A transvenous pacemaker was placed in the right ventricle through a 6-French sheath in the right femoral vein. Then, through an 8-French sheath, a Lunderquist wire was placed in the superior vena cava. This wire was used to insert a 14-French Edwards eSheath. Through this sheath, an Agilis wire was placed in an Agilis catheter. The transseptal puncture was then performed, followed by the advancement of the Agilis catheter. Through the Agilis catheter, the mitral tissue bioprosthesis was crossed with a JR4 catheter, and a wire was exchanged over the exchange length of a J wire for a pigtail.

**Figure 2:**
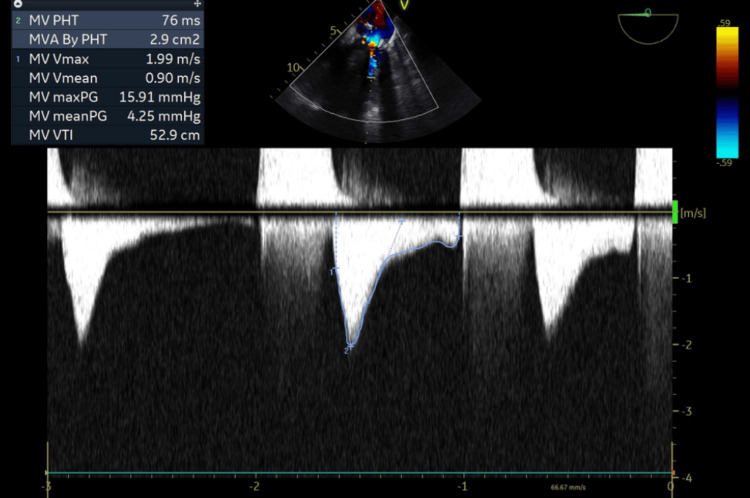
Transesophageal echocardiography showing mitral valve hemodynamics post Valve in Valve. Mean pressure gradient dropped to 4.25 mmHg.

Left Ventricle End Diastolic Pressure (LVEDP) obtained through the pigtail was measured at 16 mmHg. The pigtail was exchanged for a Lunderquist wire. A 16 mm balloon was advanced over the wire into the mitral bioprosthesis. Under fluoroscopy and TEE guidance (Video 2), balloon valvuloplasty of the mitral tissue bioprosthesis was performed. The mean gradient dropped from 30 mmHg to 13 mmHg post-valvuloplasty. The 16 mm balloon was used to perform septal dilatation. A 26 mm SAPIEN 3 valve (Edwards Lifesciences, Irvine, CA) and delivery device were then advanced through the sheath over the wire across the interatrial septum and across the mitral valve. Once in position and under rapid pacing of the heart, the new transcatheter heart valve was deployed. Once the valve deployment was completed, there was under expansion of the new valve. So, the surgical valve was fracked using a 25 mm balloon on a second rapid pacing of the heart with full inflation of the new valve (Video 3). Intra-procedural TEE showed a well-seated ViV bioprosthesis with no significant mitral regurgitation or paravalvular leak (Video 4). The mean mitral valve gradient dropped to 4 mmHG (Figure 2). There was a significant shunt in the interatrial septum, which was closed with an Amplatzer septal occluder device (Abbott, Chicago, IL) with very good results and minimal flow through the defect (Video 5). The patient tolerated the procedure well, and it was followed by an immediate favorable outcome. The patient remained on VA-ECMO support throughout the case between 3.5 and 4 L flow. After the deployment of the mitral valve, the patient's hemodynamics showed a significant improvement. Before the procedure, the patient's mean arterial pressure (MAP) was 55-60 mmHg while on maximum doses of Norepinephrine, Vasopressin, and Dobutamine. However, following the deployment, the MAP improved to 65-70 mmHg, and the patient required only minimal doses of norepinephrine. Moreover, the patient was weaned off vasopressin completely within 15-20 minutes of the mitral valve deployment. The following day, the patient was successfully weaned off norepinephrine and dobutamine.

**Video 2:** Transesophageal echocardiography showing severe prosthetic mitral valve stenosis.

**Video 3:** Successful deployment of the 26 mm SAPIEN 3 valve.

**Video 4:** Transesophageal echocardiography post ViV showing well seated ViV bioprosthesis with no significant mitral regurgitation.

**Video 5:** Fluoroscopic video showing mitral bioprosthesis (middle of the screen) and atrial septal defect closure device (left of the screen) in place.

TTE done on postoperative day one showed improved left ventricular function with an ejection fraction of 60%. After four days of the procedure, TTE showed excellent valve function with significant improvement in mitral valve hemodynamics; the mean pressure gradient dropped to 7 mmHg.

## Discussion

Transcatheter mitral valve replacement (TMVR) is a valuable treatment option in patients with failed surgical bioprostheses with a high or prohibitive surgical risk [4]. Recent studies have shown that mitral ViV procedure is associated with low complication rates and better than predicted 30-day mortality rates [4]. The transseptal mitral ViV approach, though more technically challenging than the transapical approach, is less invasive and is associated with better outcomes and shorter lengths of hospital stay [4]. The transseptal approach utilizes a small septostomy balloon and was developed to avoid complications associated with the transapical approach [5]. Currently, the US Food and Drug Administration has not approved a bioprosthetic valve specifically designed for the mitral position. However, the transcatheter heart valves used during mitral ViV were originally designed for the aortic position [4].

Bioprosthetic heart valves are described based on their external diameter. Conversely, it is the internal diameter that is important during the ViV procedure. Therefore, understanding the anatomy of the degenerated bioprosthetic mitral valve and the surrounding cardiac structures is essential before pursuing ViV intervention [6,7]. This can be accomplished using cardiac CT, TTE, and TEE. This information will help guide the heart team in using the most appropriate transcatheter heart valve size, which results in minimizing post-procedural complications such as valve migration, paravalvular leakage, or left ventricular outflow tract obstruction. The radiopaque marking within the degenerated bioprosthetic valve allows for optimal positioning of the transcatheter heart valve [3]. During the ViV procedure, our heart team decided to perform balloon valvuloplasty on the severely stenosed mitral bioprosthesis before deploying the transcatheter heart valve. This resulted in a more accessible route during the procedure. This was accomplished using a 16mm balloon.

TMVR is well-studied in stable high-risk patients with failed mitral bioprostheses [4]. However, its feasibility in patients with cardiogenic shock is unknown. There are a few case reports on the use of TMVR in patients with failed mitral bioprostheses complicated by cardiogenic shock [8]. To our knowledge, this is the third case of TMVR in a patient with failed mitral bioprosthesis complicated with cardiogenic shock requiring mechanical circulatory support (MCS) with ECMO. The first case was published in 2013 and was associated with the migration of the mitral valve into the left atrium [9]. The second case report was published in 2022. It described a patient with severe mitral valve stenosis who underwent successful TMVR under ECMO support. The patient was weaned off ECMO two days after the procedure [10]. 

In a case series of 17 patients with deteriorated mitral bioprosthesis or previous ring annuloplasty [11], Bouleti et al. reported the outcomes of transcatheter mitral ViV. The success rate (defined as the absence of procedural mortality, the correct positioning of the valve, and the absence of residual moderate or severe prosthetic regurgitation) was 82%, and complications were seen in 18% of the cases. Compared to patients who underwent elective ViV procedure, patients who underwent urgent/emergent ViV procedure had worse outcomes (one patient died, and another had migration of the valve into the left atrium). Conversely, in a case series of 19 patients with failed mitral bioprosthesis [12], a rescue procedure was performed in 12/19 (63%) patients with cardiogenic shock. Rescue mitral ViV performed through a transapical approach was associated with similar positive outcomes compared to the elective ViV procedure. However, there was no mention if any of the 12 patients with cardiogenic shock were on VA-ECMO.

One of the complications of the transseptal approach is the creation of an iatrogenic atrial septal defect. This is partly due to the large valve delivery system. This can be corrected using an atrial septal defect closure device. In our patient, we used an Amplatzer septal occluder device with very good results and minimal flow through the defect.

Data on MCS use in patients with cardiogenic shock due to severe failure of mitral bioprosthesis is scarce. Our patient had severe mitral stenosis with a mean pressure gradient of 17.2 mmHg on TTE. This resulted in limited LV filling, increased pulmonary vascular pressure and resistance, pulmonary edema, RV failure, and later, patient developed cardiogenic shock. Due to underfilling of the left ventricle, devices that rely on adequate left ventricular filling, such as the Impella device, may not be effective in cases where the reason for the cardiogenic shock is at the left atrial level. We recognize that left atrial venoarterial (LAVA) ECMO would have been our patient's optimal mechanical circulatory support method. Unfortunately, this option was not available at our hospital. VA-ECMO is another mechanical circulatory support that can be utilized to provide hemodynamic support for patients with severe mitral stenosis complicated by cardiogenic shock. VA-ECMO helps unload the left atrium and facilitates lung decongestion by diverting blood away from the lungs. VA-ECMO was used as a "bridge" to mitral valve intervention. While on VA-ECMO support, the patient successfully underwent TMVR ViV intervention. Unfortunately, the patient continued to require VA-ECMO due to persistent hypoxia, which was caused by diffuse alveolar hemorrhage and hospital-acquired pneumonia. We discussed switching him to venovenous ECMO with the family, but they decided to transition him to hospice.

## Conclusions

The transcatheter transseptal mitral valve-in-valve procedure is a practical intervention in critically ill patients due to a failed mitral bioprosthesis. Mechanical circulatory support using VA-ECMO should not preclude these patients from undergoing this life-saving intervention. Long-term follow-up studies are necessary to evaluate the intervention's outcomes in critically ill patients.

## References

[1] Vemulapalli S, Grau-Sepulveda M, Habib R, Thourani V, Bavaria J, Badhwar V (2019). Patient and hospital characteristics of mitral valve surgery in the United States. JAMA Cardiol.

[2] Mehaffey HJ, Hawkins RB, Schubert S (2018). Contemporary outcomes in reoperative mitral valve surgery. Heart.

[3] Walther T, Falk V, Dewey T (2007). Valve-in-a-valve concept for transcatheter minimally invasive repeat xenograft implantation. J Am Coll Cardiol.

[4] Whisenant B, Kapadia SR, Eleid MF (2020). One-year outcomes of mitral valve-in-valve using the Sapien 3 transcatheter heart valve. JAMA Cardiol.

[5] Eleid MF, Cabalka AK, Williams MR (2016). Percutaneous transvenous transseptal transcatheter valve implantation in failed bioprosthetic mitral valves, ring annuloplasty, and severe mitral annular calcification. JACC Cardiovasc Interv.

[6] Wilbring M, Alexiou K, Tugtekin SM, Sill B, Simonis G, Matschke K, Kappert U (2013). Transcatheter valve-in-valve therapies: patient selection, prosthesis assessment and selection, results, and future directions. Curr Cardiol Rep.

[7] Wang DD, Eng MH, Greenbaum AB (2018). Validating a prediction modeling tool for left ventricular outflow tract (LVOT) obstruction after transcatheter mitral valve replacement (TMVR). Catheter Cardiovasc Interv.

[8] Rufian-Andujar S, Iftikhar O, Salinger M, Saucedo J, Feldman T, Guerrero M (2018). Transseptal transcatheter mitral valve-in-valve for treatment of severe mitral regurgitation in failed bioprosthesis complicated with cardiogenic shock: Case report and review of the literature. Cardiovasc Revasc Med.

[9] Fassa AA, Himbert D, Brochet E (2013). Emergency transseptal transcatheter mitral valve-in-valve implantation. EuroIntervention.

[10] Clark RM, Bruoha S, Milwidsky A (2022). Venoarterial extracorporeal membrane oxygenation to facilitate transcutaneous mitral valve replacement in critical mitral stenosis. JACC Case Rep.

[11] Bouleti C, Fassa AA, Himbert D (2015). Transfemoral implantation of transcatheter heart valves after deterioration of mitral bioprosthesis or previous ring annuloplasty. JACC Cardiovasc Interv.

[12] Elmously A, Worku B, Gray KD, Salemi A (2018). Mitral valve-in-valve implantation as an elective or rescue procedure in high-risk patients. Ann Thorac Surg.

